# Quantifying Benefit-Risk Trade-Offs Toward Prophylactic Treatment Among Adult Patients With Hemophilia A in China: Discrete Choice Experiment Study

**DOI:** 10.2196/45747

**Published:** 2023-07-26

**Authors:** Limin Wang, Shimeng Liu, Shan Jiang, Chaofan Li, Liyong Lu, Yunhai Fang, Shunping Li

**Affiliations:** 1 Centre for Health Management and Policy Research School of Public Health，Cheeloo College of Medicine Shandong University Jinan China; 2 NHC Key Laboratory of Health Economics and Policy Research Shandong University Jinan China; 3 Centre for Health Preference Research Shandong University Jinan China; 4 School of Public Health Fudan University Shanghai China; 5 NHC Key Laboratory of Health Technology Assessment Fudan University Shanghai China; 6 Macquarie University Centre for the Health Economy Macquarie Business School and Australian Institute of Health Innovation Macquarie University Sydney Australia; 7 Shandong Hemophilia Treatment Center Shandong Blood Center Jinan China

**Keywords:** benefit-risk assessment, discrete choice experiment, hemophilia A, patient preference, prophylactic treatment

## Abstract

**Background:**

Hemophilia A is a chronic condition that requires meticulous treatment and management. Patient preferences for prophylactic treatment can substantially influence adherence, outcomes, and quality of life, yet these preferences remain underexplored, particularly in China.

**Objective:**

This study aimed to investigate the preferences for prophylactic treatment among Chinese adult patients with hemophilia A without inhibitors, considering clinical effectiveness, side effects, dosing mode, and dosing frequency.

**Methods:**

A discrete choice experiment was used to elicit patient preferences for prophylactic treatment of hemophilia. The study was conducted across 7 provinces in China with socioeconomic and geographical diversity. Subgroup analysis was performed according to education level, geographic location, and treatment type, alongside the exploration of benefit-risk trade-offs.

**Results:**

A total of 113 patients completed the discrete choice experiment questionnaire, and we included 102 responses for analysis based on predetermined exclusion criteria. The study found that patients prioritized reducing annual bleeding times and avoiding the risk of developing inhibitors over treatment process attributes. Subgroup analysis revealed that lower-educated patients and those from rural areas attached more importance to the dosing mode, likely due to barriers to self-administration. Patients demonstrated a clear understanding of benefit-risk trade-offs, exhibiting a willingness to accept an increased risk of developing inhibitors for improved clinical outcomes.

**Conclusions:**

This study provides valuable insights into the preferences of patients with hemophilia A for prophylactic treatment in China. Understanding these preferences can enhance shared decision-making between patients and clinicians, fostering personalized prophylactic treatment plans that may optimize adherence and improve clinical outcomes.

## Introduction

Hemophilia, an infrequent hemorrhagic disorder, is primarily triggered by the absence or deficiency of specific coagulation factors in the blood [1]. According to the World Federation of Hemophilia’s 2018 global survey, China was found to have the second highest incidence of confirmed hemophilia cases globally [2]. With a prevalence of 2.73 per 100,000 individuals, hemophilia A (ie, classical hemophilia) constituted more than 80% of these instances [3]. The ensuing joint deformities and disabilities resulting from bleeding into tissues, joints, and muscles critically impair the quality of life for these patients [1].

Factor replacement therapy, classified into on-demand treatment (administered during bleeding episodes) and prophylactic treatment (routine administration to prevent bleeding), is the prevailing treatment modality [4]. However, the development of inhibitors, or an immune response to the therapy, constitutes the most severe adverse effect of this approach [5]. Patients with hemophilia A who have developed inhibitors undergo hemostasis and the removal of inhibitors [4]. In contrast, for patients with hemophilia A devoid of inhibitors, prophylactic treatment remains the optimal care strategy [4].

China has actively pursued and advocated prophylactic treatment measures appropriate to its national context, fostering comprehensive health management of patients with hemophilia [6,7]. Nevertheless, a key impediment remains low adherence to prophylactic treatment. Alarmingly, the rates of prophylactic treatment and treatment compliance are substantially lower in China compared to developed nations (4.1% vs 36.8% and 6.2% vs 40.5%, respectively) [8]. Previous studies have indicated that various factors, such as age, health status, annual bleeding times, infusion methods, dosing regimens, and cost, significantly influence the uptake of prophylactic treatment among adult patients with hemophilia [9,10]. Patients with hemophilia A receiving prophylactic treatment experience a reduction in annual bleeding times, yet they face an associated risk of developing inhibitors. This makes establishing a benefit-risk balance challenging. Therefore, assessments of potential benefits and harms can aid stakeholders, regulators, health technology assessors, and health professionals in understanding and communicating treatment-risk balance, ultimately enhancing adherence to prophylactic treatment [11].

The discrete choice experiment (DCE) is an established stated preference method, extensively used to quantify patients’ preferences concerning health care [12-16]. Several DCEs have been conducted to elicit the treatment preferences of patients with hemophilia [17-23]. However, evidence regarding the preference for prophylactic treatment among adult patients with hemophilia A without inhibitors, particularly in terms of benefit-risk assessment, remains sparse in mainland China. This study endeavors to address this research gap by using a DCE as a patient decision aid. It aims to identify the preferences of adult patients with hemophilia A without inhibitors in China concerning the risk and benefit attributes when selecting prophylactic treatment.

## Methods

### Overview

The DCE is a renowned tool used to simulate the influence of diverse attributes of a service or commodity on individual preference [15,16]. In the scope of a DCE, participants are presented with the task of deciding between 2 or more hypothetical incentive scenarios, differentiated by several relevant dimensions, termed *attributes*. These attributes present varied configurations, referred to as *levels*, across the proposed alternatives [15].

### Identification of Attributes and Levels

The onset of the DCE involved pinpointing relevant attributes. In accordance with research guidelines, a combination of qualitative and quantitative methods was applied to derive attributes and levels [24]. A total of 7 attributes were ascertained through literature reviews and expert consultations, including the annual bleeding times [21-23,25-28], the risk of developing inhibitors [17-21,27], dosing frequency [18,19,21,23,26-30], dosing mode [26,27,30], storage temperature [21,29], dosage form [18,20,22,26], and the risk of virus infection [17-20]. Following face-to-face patient interviews and expert focus group discussions, 3 attributes (storage temperature, dosage form, and virus infection risk) were dismissed. Due to its complexity, cost was also discarded as a function of individual factors such as weight, the type and specification of medication, and regional health insurance reimbursement rates (varying from 40% to 90%) [31]. Consequently, 4 attributes were selected for inclusion in this study: annual bleeding times, dosing frequency, dosing mode (these 3 represent benefits), and the risk of developing inhibitors (this represents risk). The levels of the 4 DCE attributes were sourced from drug instructions and clinical prophylactic treatment studies [5,32-35]. The chosen attributes and their corresponding levels are detailed in Table 1.

**Table 1:** Attributes and levels for discrete choice experiment questions.

Attributes	Levels
Annual bleeding times	0 times per year 6 times per year 12 times per year
Risk of developing inhibitors	0% 2% 4%
Dosing frequency	1 time per week 2 times per week 3 times per week
Dosing mode	Intravenous drip Intravenous push Subcutaneous

### Questionnaire and DCE Instrument Design

**Figure 1:**
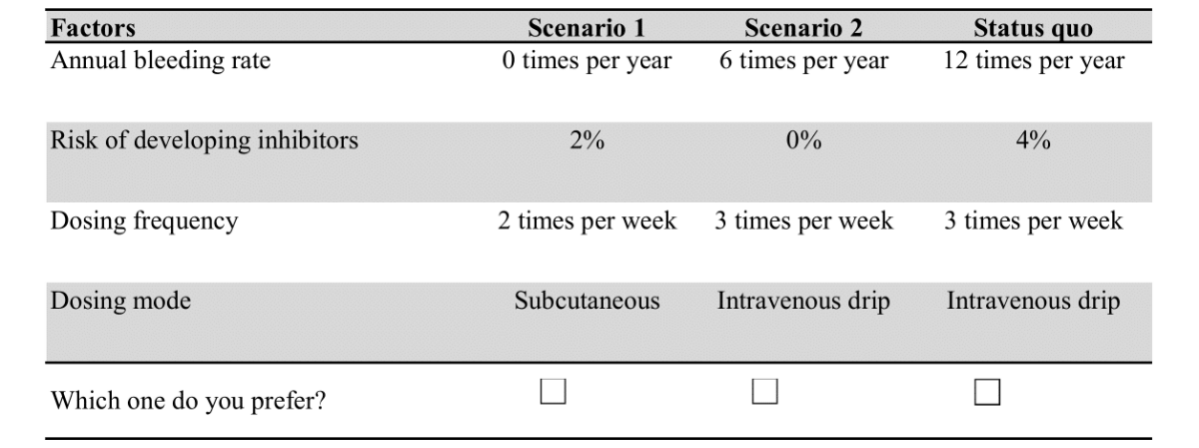
An example question in the discrete choice experiment (translated version).

The D-optimal design approach was used to produce 18 choice tasks in SAS (version 9.2; SAS Institute). To alleviate cognitive load, these tasks were divided into 2 blocks. To reflect a more authentic clinical decision-making context, a status quo option was introduced in each choice task. An example of a DCE question is provided in Figure 1.

The questionnaire encapsulated an introduction to the research background, attribute definitions, 9 DCE choice tasks, and sociodemographic inquiries. Preceding the official choice tasks, a practice task acclimated respondents to the forthcoming tasks. A question was repeated in each block to validate the internal consistency of the survey. The sociodemographic information gathered included sex, age, education level, employment status, and geographic location.

To ensure respondents comprehended the definition of the risk of developing inhibitors, they were posed a question regarding their understanding of risk percentages [36]. For instance, given a 4% risk of developing inhibitors, respondents were asked to determine how many patients out of 100 would potentially develop inhibitors. The successful answer to this question was a prerequisite for proceeding with the DCE choice tasks.

A pilot study was undertaken among adult patients with hemophilia A without inhibitors at the Shandong Province Hemophilia Treatment Center. The pilot sought to evaluate the understandability, acceptability, and validity of the questionnaire, resulting in revisions to the language and layout.

### Sample Selection

This study’s sample size was guided by a rule of thumb frequently used in DCE studies that the sample size should comprise no fewer than 75 respondents [37].

Participants were chiefly sourced from Shandong, Hebei, and Henan, which have the largest population of patients with hemophilia A registered with the Hemophilia Treatment Center Collaboration Network of China [38]. Additional patients were recruited from 4 other provinces: Jiangsu, Hubei, Hunan, and Chongqing. The inclusion criteria comprised a hemophilia A diagnosis, no inhibitors present at the time of recruitment, being aged 18 years or older, and receiving treatment for more than 50 exposure days. Exposure days refer to the cumulative number of days coagulation factor VIII was injected, which influences the likelihood of producing an inhibitor.

### Data Collection

Formal data collection occurred between December 2021 and March 2022. To ensure data quality, we sent the electronic questionnaire to respondents first and then conducted the interview over the telephone in a one-on-one manner. The interviewers answered respondents’ questions. Through a warm-up section, the interviewers trained the respondents to familiarize themselves with the DCE choice tasks and guarantee they comprehended the whole questionnaire.

### Model Specification

A mixed logit model was used to analyze the choice data in Stata (version 15.1; StataCorp), estimated through the simulated maximum likelihood approach [39,40]. The mixed logit model included an alternative-specific constant (ASC) indicative of the utility generated by the status quo option in comparison to non–status quo options. We hypothesized that the parameters of attribute levels would conform to a normal distribution. Parameter estimations were derived relative to the reference level within each attribute. The average preference value, termed *part-worth utility*, and the variability of the preference value among patients with hemophilia were represented by the mean and SD of a parameter, respectively. Model fit was determined by the Akaike information criterion (AIC), Bayesian information criterion (BIC), and log-likelihood ratio. To ensure the reliability of parameter estimates, we iteratively estimated the mixed logit model by incrementally increasing the number of random draws by 500, commencing with 50 draws. Estimation stability was achieved at 2500 draws, producing our final estimates [41].

We excluded patients who failed the consistency test or always chose the options on the left or right in all the choice tasks (ie, position bias) [15]. We also conducted a sensitivity check to examine whether the exclusion would significantly affect the results of the mixed logit model.

### Dominant Preference Examination

Dominant preference refers to the phenomenon whereby a respondent’s choices are dictated by a single attribute, resulting in decisions that consistently favor the alternative with a superior level of 1 attribute in a choice task [42]. Such respondents avoid making trade-offs between attributes, impeding the analysis of relative importance between attributes [42]. We used nonparametric [42] and parametric [43] approaches to test for dominant preference. The nonparametric approach evaluated if respondents’ choices exhibited a pattern, that is, if the selected alternatives for the 7 tasks were invariably consistent with the alternatives with a higher level of a certain attribute in comparison to the other alternative in all 7 questions. The parametric approach compared the estimated coefficients of attribute levels between a full model encompassing all attributes and a reduced model containing only 1 attribute. Notable discrepancies between the coefficients indicated the presence of a dominant preference driven by that attribute. We iteratively inserted each attribute into the reduced model and compared the model estimates with the full model.

### Attribute Relative Importance

The relative importance of each attribute was computed using the mixed logit model estimates through a widely used rescaling method [16,44]. The relative importance of each attribute was determined by dividing the range of coefficients within the attribute by the sum of all attribute ranges, which was subsequently rescaled to a 1-100 range. The highest value denoted the attribute perceived as most important by the respondents.

### Interaction

A meticulous examination of all potential interactions between the characteristics of respondents and attribute levels was carried out using the multinomial logit model (MNL) [15]. Interaction terms were selected using a backward selection method based on the contribution of each term to model fit. The log-likelihood ratio test was used to compare the model specifications with a reduced model with 1 interaction term removed. If the removed term significantly influenced the model fit, the term was retained. Following the identification of interaction terms, we simulated a mixed logit model that incorporated the interactions to quantify the preference values assigned to the interaction terms by respondents.

### Maximum Acceptable Risk

We quantified patients’ tolerance for the risk of developing inhibitors in exchange for improvements in other attributes. The outcome was termed the maximum acceptable risk (MAR) by patients. The MAR gauged the benefit-risk ratio that patients were willing to accept in terms of the trade-off between benefit and risk, given that the utility remained constant [45].

### Subgroup Analysis

We conducted subgroup analyses by comparing the relative importance of attributes between different groups of respondents using individual-level preferences. We applied a mixed logit model to the variables used to categorize respondents, which encompassed education level, geographic location, and treatment type.

### Ethics Approval

Ethical approval for this study was granted by the Center for Health Management and Policy Research, Shandong University (ECSHCMSDU20211102).

## Results

### Pilot Study

This pilot study incorporated a cohort of 15 patients, all of whom successfully completed the study. The derived model estimates were in alignment with our theoretical anticipations in terms of their coefficient signs and priority order, thereby affirming that the participants comprehended the choice tasks and that the quantity of questions was manageable. The mean time taken to conclude the study was approximately 15 minutes.

### Patient Characteristics

We invited 140 patients who were eligible for inclusion, and 122 consented to participate (a response rate of 87%), of whom 113 completed the survey (a completion rate of 93%). On average, participants took 14.60 (SD 5.41) minutes to complete the questionnaire. Among those who completed the questionnaire, 10 participants did not pass the consistency test, while 1 participant exhibited position bias (ie, always choosing the left or right options); hence, we excluded them from further analysis. Thus, a total of 102 participants were included in the final analysis.

The demographic profile of the participants included in the analysis is presented in Table 2. The majority of patients were male (100/102, 98%), possessed an education level equivalent to high school or higher (54/102, 52.9%), were single (59/102, 57.8%), and resided in townships or rural areas (66/102, 64.7%). Most patients were diagnosed with severe hemophilia A (58/102, 56.9%) and received on-demand treatment (56/102, 54.9%).

**Table 2:** Sociodemographic characteristics of patients included in the analysis.

Characteristic		Patients included in the study (n=102)
Age (in years), mean (SD)		33.8 (7.5)
**Sex, n (%)**		
	Male	100 (98)
	Female	2 (1.9)
**Education level, n (%)**		
	Middle school or below	48 (47.1)
	High school or above	54 (52.9)
**Marital status, n (%)**		
	Unmarried	59 (57.8)
	Married	41 (40.2)
	Divorced	2 (2)
**Occupation status, n (%)**		
	Unemployed	42 (41.2)
	Working full time	25 (24.5)
	Working part time	11 (10.8)
	Self-employed	9 (8.8)
	Student	14 (13.7)
	Retired	1 (1)
**Geographic location, n (%)**		
	Urban	36 (35.3)
	Rural	66 (64.7)
**Severity of hemophilia A, n (%)**		
	Mild	3 (2.9)
	Moderate	41 (40.2)
	Severe	58 (56.9)
**Treatment type, n (%)**		
	On-demand	56 (54.9)
	Prophylaxis	41 (40.2)
	Nonstandard prophylaxis	5 (4.9)

### Patients’ Preferences

Table 3 presents the mixed logit model results. The constant representing the status quo option was not statistically significant, indicating patients expressed no preference for the status quo compared with nonstatus quo alternatives.

Respondents expressed a higher likelihood of accepting either 0 or 6 instances of bleeding annually, as opposed to 12. Utility declined as the risk of developing inhibitors increased. In terms of dosing frequency, patients demonstrated a preference for 1 or 2 doses per week over 3. With respect to the mode of administration, intravenous pushes and subcutaneous injections were preferred over intravenous drips.

We did not identify the presence of dominant preferences through either parametric and nonparametric approaches. A sensitivity analysis was conducted by including the 11 patients who failed the internal validity test or exhibited position bias (Table S1 in Multimedia Appendix 1). We found no significant differences between the 2 models—one including and the other excluding these 11 patients—indicating that the exclusion did not affect the findings.

**Table 3:** Mixed logit model results.

				β		*P* value		SE		SD		*P* value		SE
Constant				0.72		.373		0.80		3.80		<.001		0.76
**Annual bleeding times (** **reference level** **12 per year)**														
		6 per year	1.80		<.001		0.25		0.00		.99		0.38	
		0 per year	3.64		<.001		0.39		1.49		<.001		0.31	
Risk of developing inhibitors				−0.79		<.001		0.39		0.54		<.001		0.09
**Dosing frequency (** **reference level** **3 times per week)**														
		2 times per week	1.20		<.001		0.24		0.05		.88		0.32	
		1 time per week	1.58		<.001		0.25		0.01		.98		0.38	
**Dosing mode (** **reference level** **intravenous drip)**														
	Intravenous push			0.74		<.001		0.19		0.07		.84		0.33
	Subcutaneous			1.35		<.001		0.29		1.95		<.001		0.34

**Attribute Relative Importance**

The frequency of annual bleeding events emerged as the most significant attribute from the perspective of the participants, with the attribute relative importance (ARI) score at 100. The risk of developing inhibitors ranked second in importance (ARI=87), while dosing frequency (ARI=43) and dosing mode (ARI=37) were considered comparatively less critical.

### Interaction

The selection of interaction terms identified a significant interaction between the status-quo constant and the education level of participants (Table S2 in Multimedia Appendix 1). The positive preference value suggested that respondents with a higher level of education (ie, high school or above) were more likely to opt for the status quo alternative than those with a lower educational level (ie, middle school or below).

### Subgroup Analysis

Subgroup analysis was performed based on variables such as education level, geographic location, and treatment type. Patients with lower education levels (middle school or below) considered the dosing mode more important than the dosing frequency. Conversely, patients with a higher education level (high school or above) placed greater importance on dosing frequency compared to dosing mode (Figure S1 in Multimedia Appendix 1). A similar contrast was observed between urban and rural respondents (Figure S2 in Multimedia Appendix 1). While urban respondents emphasized the dosing frequency over the dosing mode, rural respondents held the opposite view. The comparison of patients undergoing prophylaxis treatment versus those receiving on-demand treatment revealed a common preference pattern; both groups viewed dosing frequency as more important than dosing mode (Figure S3 in Multimedia Appendix 1).

### Benefit-Risk Trade-Off

**Figure 2:**
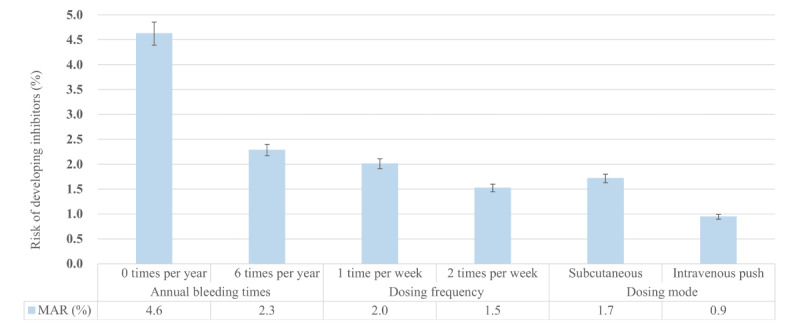
Benefit-risk trade-off and maximum acceptable risk. MAR: maximum acceptable risk.

The results pertaining to MAR are depicted in Figure 2. For specified improvements in treatment attributes such as annual bleeding frequency, dosing frequency, and dosing mode, the MAR indicates the highest level of risk related to developing inhibitors that patients would tolerate, thereby hinting at patients’ willingness to accept a trade-off between potential benefits and risks.

Patients were willing to accept an increase in the risk of developing inhibitors (MAR=4.6% for reducing annual bleeding instances from 12 to 0). They would also tolerate an increased risk of 2.3% for reducing the frequency of bleeding from 12 to 6 times per year. Furthermore, an increased risk of 2% would be acceptable to patients if the dosing frequency was decreased from 3 times a week to once a week, while an increased risk of 1.5% would be acceptable if the dosing frequency was reduced from 3 to 2 times a week. In terms of dosing mode, patients would tolerate a risk increase of 1.7% if the dosing model shifted from intravenous drip to subcutaneous injection and a risk increase of 0.9% if the dosing model was altered from intravenous drip to intravenous push.

## Discussion

### Overview

This is the first study investigating the preferences of patients with hemophilia A without inhibitors regarding prophylactic treatment by performing a DCE in China. We examined 4 attributes associated with prophylactic treatment, with results indicating patients placed higher value on clinical effectiveness (ie, reduction in annual bleeding times) and side effects (ie, risk of developing inhibitors) compared to aspects of the treatment process (ie, dosing frequency and mode). We also found that patients were willing to accept an increase of 4.6% in the risk of developing inhibitors for the reduction of bleeding from 12 times per year to 0 times per year.

This study showed that patients attach paramount importance to the reduction of annual bleeding times, an outcome consistent with previous investigations [21,25,27]. Our findings suggest that alterations in the mode and frequency of administration exert a relatively minimal influence on patients’ preferences. In contrast, a previous study reported dissimilar results by using conjoint analysis to ascertain patient and parental preferences for prophylactic treatment [26]. Their study indicated that the frequency of prophylactic administration was deemed the most crucial attribute, followed by the annual bleeding times. This discrepancy could be attributed to differences in the standard of treatment across various countries. China primarily uses low-dose prophylaxis, which has comparatively less control over bleeding than standard-dose prophylaxis [46]. The aforementioned study, conducted in Australia, Britain, and the United States, demonstrated that reduced instances of breakthrough bleeds during prophylaxis (including 0, 1, 2, and 3 times per year) exerted a lesser influence on patient preferences. Given the current status of prophylactic treatment in China, it is plausible to propose that patients may favor a treatment regimen with enhanced bleeding control, which has implications for the development of personalized prophylactic treatment protocols [47].

### Individual-Level Factors

Subgroup analysis revealed that patients with lower education levels prioritized the dosing mode. Patients residing in townships or rural areas also exhibited higher sensitivity toward the dosing mode compared to their urban counterparts. This could be attributed to the barriers they face in the self-administration of prophylactic infusions. Unlike typical oral or inhaled medications, coagulation factors require injection, necessitating specialized self-care skills or medical provider support. Self-injection therapy for patients with hemophilia remains challenging [48], particularly for patients with a lower level of education, who are less likely to master self-injection techniques. Patients in townships or rural areas often have a lower quality of life compared to those in urban areas [49]. Thus, it is essential to focus on self-management treatment services for patients with low education levels and those in rural areas. Such services could include intravenous injection training programs and consultation and guidance services [48].

### Benefit-Risk Trade-Off

This study illustrated that patients demonstrated clear awareness of the benefit-risk assessment. The results suggested that patients were willing to accept a substantial risk of developing inhibitors for reducing the annual bleeding times, but were only willing to accept a minor risk alteration when changing drug administration from intravenous drip to intravenous push. The risk of developing inhibitors could be controlled by the treatment strategy [50,51]. Particularly, a heterogeneity analysis revealed that patients undergoing prophylactic treatment were more attuned to the risk of developing inhibitors. We propose that physicians must judiciously balance the trade-offs between bleeding and the risk of developing inhibitors when recommending prophylactic treatment for patients. This is of particular importance when optimal control of bleeding is achieved, as the risks of developing inhibitors may exceed the maximum acceptable range for the patient.

### Limitations

This study fills a significant gap in our understanding of treatment preferences for people with hemophilia in mainland China. However, certain limitations persist. There could be potential sampling bias as we did not use random sampling. Nonetheless, to enhance the representativeness of our results, we examined the prophylactic preferences of adult patients with hemophilia A without inhibitors across 7 regions of China, accounting for geographical diversity and varied economic development status. Moreover, DCE, being a hypothetical choice scenario, cannot accurately track actual choice behavior. Therefore, to mitigate the impact of this potential discrepancy, we ensured the attribute levels were scientifically valid and realistic in the preliminary stage, thereby providing respondents with a credible profile for comparison and evaluation.

### Conclusions

Our study’s findings significantly contribute to understanding the preferences of adult patients with hemophilia A regarding prophylactic treatment. Acknowledging that patients will perceive trade-offs for the 4 attributes differently can enhance the dialogue between patients and clinicians on the risks and benefits of various prophylactic treatment modalities.

## Appendix

Multimedia Appendix 1 Supplementary material for conditional logit models, results of mixed logit model with main effects and interactions, attribute relative importance perceived by high-education respondents vs low-education respondents, attribute relative importance perceived by urban respondents vs rural respondents, and attribute relative importance perceived by respondents receiving prophylactic treatment vs respondents receiving on-demand treatment.

## Abbreviations

AIC: Akaike information criterion
ARI: attribute relative importance
ASC: alternative-specific constant
BIC: Bayesian information criterion
DCE: discrete choice experiment
MAR: maximum acceptable risk
MNL: multinomial logit model
