# Supplementary material for: Quantifying Benefit-Risk Trade-Offs Toward Prophylactic Treatment Among Adult Patients With Hemophilia A in China: Discrete Choice Experiment Study
Source: JMIR Public Health Surveill. 2023 Jul 26;9:e45747. doi: 10.2196/45747 (PMC10413247; doi:10.2196/45747)
Supplement: Multimedia Appendix 1 [file publichealth_v9i1e45747_app1.docx]

**Supplementary Materials and Results**

Table S1. Conditional logit models: Full sample (n=113) vs. sample including patients who passed consistency test (n=102).

|  | N=113 | | N=102 | |
| --- | --- | --- | --- | --- |
|  | Mean | P-value | Mean | P-value |
| Constant | 1.041 | .002 | 1.122 | .003 |
|  |  |  |  |  |
| Annual bleeding rate (ref. 12 per year) | | | |  |
| 0 times per year | 1.841 | <.001 | 1.949 | <.001 |
| 6 times per year | 0.946 | <.001 | 0.987 | <.001 |
|  |  |  |  |  |
| Risk of developing inhibitors (ref. 4%) | |  |  |  |
| 0% | 1.643 | <.001 | 1.764 | <.001 |
| 2% | 0.867 | <.001 | 0.973 | <.001 |
|  |  |  |  |  |
| Dosing frequency (ref. 3 times per week) | | | |  |
| 1 time per week | 0.729 | <.001 | 0.734 | <.001 |
| 2 times per week | 0.479 | <.001 | 0.487 | <.001 |
|  |  |  |  |  |
| Dosing mode (ref. Intravenous drip) | | | |  |
| Subcutaneous | 0.796 | <.001 | 0.791 | <.001 |
| Intravenous push | 0.456 | <.001 | 0.511 | <.001 |

Note: ref, reference.

Table S2. Results of mixed logit model with main effects and interactions.

|  | β | SE | P Value | SD | P Value |
| --- | --- | --- | --- | --- | --- |
| Constant | -0.320 | 0.941 | .734 | 3.180 | <.001 |
|  |  |  |  |  |  |
| Annual bleeding times |  |  |  |  |  |
| 12 times per year | 0 | ― | ― | ― | ― |
| 6 times per year | 1.774 | 0.2445 | <.001 | 0.033 | .923 |
| 0 times per year | 3.571 | 0.371 | <.001 | 1.456 | <.001 |
|  |  |  |  |  |  |
| Risk of developing inhibitors | -0.785 | 0.097 | <.001 | 0.558 | <.001 |
|  |  |  |  |  |  |
| Dosing frequency |  |  |  |  |  |
| 3 times per week | 0 | ― | ― | ― | ― |
| 2 times per week | 1.167 | 0.240 | <.001 | 0.035 | .907 |
| 1 time per week | 1.570 | 0.249 | <.001 | 0.053 | .894 |
|  |  |  |  |  |  |
| Dosing mode |  |  |  |  |  |
| Intravenous drip | 0 | ― | ― | ― | ― |
| Intravenous push | 0.731 | 0.190 | <.001 | 0.060 | .86 |
| Subcutaneous | 1.341 | 0.287 | <.001 | 1.927 | <.001 |
|  |  |  |  |  |  |
| Interaction |  |  |  |  |  |
| ASC_edu_int | 2.200 | 0.934 | .018 | 1.115 | .197 |

Note: ASC_edu_int, interaction term between the alternative-specific constant (ASC) of status-quo alternative and the education level of respondents.


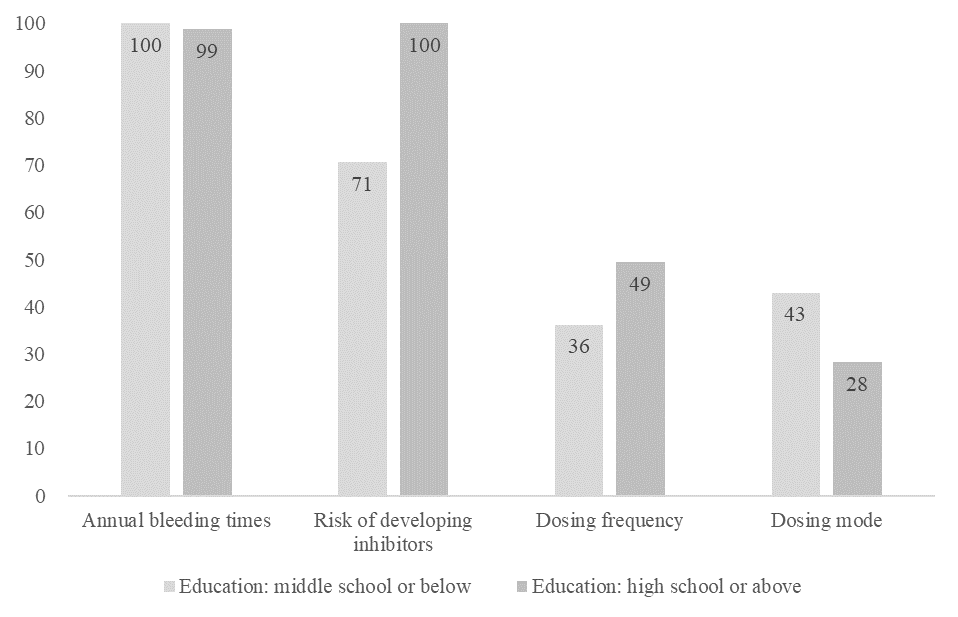


Figure S1. Attribute relative importance perceived by high-education respondents vs. low-education respondents.


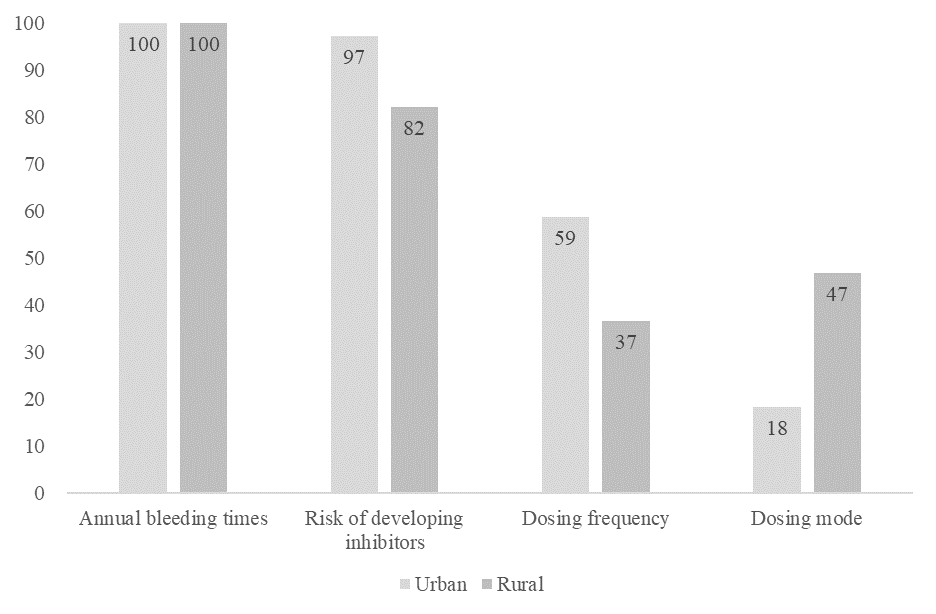


Figure S2. Attribute relative importance perceived by urban respondents vs. rural respondents.


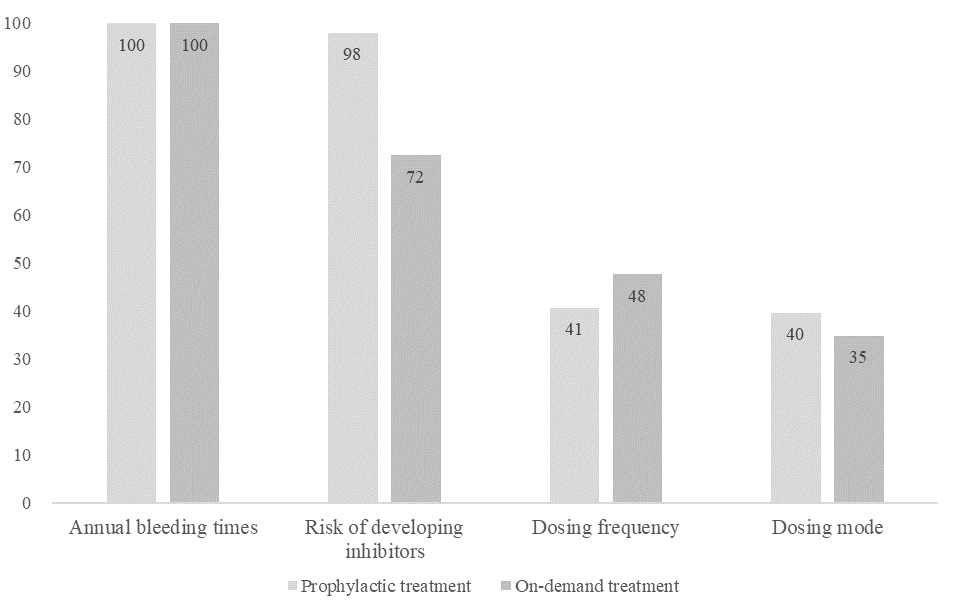


Figure S3. Attribute relative importance perceived by respondents receiving prophylactic treatment vs. respondents receiving on-demand treatment.
